# Prone positioning in mechanically ventilated patients with severe acute respiratory distress syndrome and coronavirus disease 2019

**DOI:** 10.1111/aas.13741

**Published:** 2020-11-22

**Authors:** Helena Gleissman, Anders Forsgren, Elisabeth Andersson, Elin Lindqvist, Adam Lipka Falck, Maria Cronhjort, Martin Dahlberg, Mattias Günther

**Affiliations:** ^1^ Department of Women's and Children's Health Stockholm Sweden; ^2^ Department of Clinical Science and Education Södersjukhuset Section for Anesthesiology and Intensive Care Karolinska Institutet Stockholm Sweden

**Keywords:** acute respiratory distress syndrome, COVID‐19, intensive care, oxygenation, prone position, responders

## Abstract

**Background:**

The management of COVID‐19 ARDS is debated. Although current evidence does not suggest an atypical acute respiratory distress syndrome (ARDS), the physiological response to prone positioning is not fully understood and it is unclear which patients benefit. We aimed to determine whether proning increases oxygenation and to evaluate responders.

**Methods:**

This case series from a single, tertiary university hospital includes all mechanically ventilated patients with COVID‐19 and proning between 17 March 2020 and 19 May 2020. The primary measure was change in PaO_2_:FiO_2_.

**Results:**

Forty‐four patients, 32 males/12 females, were treated with proning for a total of 138 sessions, with median (range) two (1‐8) sessions. Median (IQR) time for the five sessions was 14 (12‐17) hours. In the first session, median (IQR) PaO_2_:FiO_2_ increased from 104 (86‐122) to 161 (127‐207) mm Hg (*P* < .001). 36/44 patients (82%) improved in PaO_2_:FiO_2_, with a significant increase in PaO_2_:FiO_2_ in the first three sessions. Median (IQR) FiO_2_ decreased from 0.7 (0.6‐0.8) to 0.5 (0.35‐0.6) (<0.001). A significant decrease occurred in the first three sessions. PaO_2_, tidal volumes, PEEP, mean arterial pressure (MAP), and norepinephrine infusion did not differ. Primarily, patients with PaO_2_:FiO_2_ approximately < 120 mm Hg before treatment responded to proning. Age, sex, BMI, or SAPS 3 did not predict success in increasing PaO_2_:FiO_2_.

**Conclusion:**

Proning increased PaO_2_:FiO_2_, primarily in patients with PaO_2_:FiO_2_ approximately < 120 mm Hg, with a consistency over three sessions. No characteristic was associated with non‐responding, why proning may be considered in most patients. Further study is required to evaluate mortality.

**Editorial Comment**

Adult respiratory distress syndrome in COVID‐19 patient presents a major clinical management challenge. In the analysis of this single‐center case series, short‐term clinical responses to the therapeutic interventions are presented, with a focus on prone position and non‐responsiveness.

## 1 INTRODUCTION

Coronavirus disease 2019 (COVID‐19) is a pandemic affecting more than 39 million people worldwide and carrying a case fatality rate of 3% as of October 2020.^1^ A substantial proportion of patients with COVID‐19 develop severe respiratory failure and require mechanical ventilation, often fulfilling the criteria for acute respiratory distress syndrome (ARDS).^2^ The management of ARDS secondary to COVID‐19 is challenging and debated. Early reports suggested the likelihood of an atypical pathophysiology to explain the pulmonary and systemic manifestations such as the presence of severe hypoxemia with preserved pulmonary mechanics.^3^ Some patients with COVID‐19 ARDS present with low PaO_2_:FiO_2_ ratios despite preserved compliance, which differs from classic ARDS.^4^, ^5^ However, emerging evidence indicate that the respiratory system mechanics of patients with ARDS, with or without COVID‐19, are broadly similar, advocating standard evidence‐based management for ARDS.^6^ Prone positioning is considered as one of the most effective strategies for patients with severe ARDS,^7^ with improvement in oxygenation attributed to perfusion redistribution, more homogeneous inflation‐ventilation, better lung/thoracic shape mismatch, and improved chest wall elastance.^8^ While prone positioning is currently used in up to 76% of mechanically ventilated COVID‐19 patients,^2^, ^9^ the physiological response to proning has not been evaluated in detail, and it is not fully known whether proning leads to improved PaO_2_:FiO_2_ similarly to non‐COVID‐19 ARDS, or which patients benefit from the treatment. The aims of this case series are to describe the respiratory and circulatory effects of prone positioning in mechanically ventilated patients with COVID‐19 ARDS in the ICU, to evaluate which patients may respond to proning, and to investigate whether oxygenation improves after repeated proning.

## 2 METHODS

We retrospectively reviewed all mechanically ventilated adult patients who were treated with prone positioning in the ICU at Södersjukhuset, a tertiary university hospital, between 17 March 2020 and 19 May 2020. Respiratory parameters were collected at four times: 1 hour before proning, 1 hour after the start of proning, 1 hour before return to supine, and 1 hour after return to supine. Follow‐up was conducted at 30 days from first proning to determine how many patients were still admitted to the ICU, discharged from the ICU, or deceased. All continuous data are presented as medians with interquartile range (IQR). The primary endpoint was change in PaO_2_:FiO_2_. A power analysis was not performed due to the novelty of the disease during the observation period. Continuous data were compared using Wilcoxon signed rank test, comparing the times 1 hour before proning and 1 hour before return to supine. Analyses were performed using GraphPad Prism (v 8.4.1). Two‐sided *P*<.05 defined statistical significance. An ordinal multivariable regression model of the change in PaO_2_:FiO_2_ as a response to proning with continuous covariables modeled as 4‐knot restricted cubic splines was applied using R (v 3.5.1). The predicted mean effect of initial PaO_2_:FiO_2_ at the median of the other covariables was used. The study was approved by the Swedish Ethical Review Authority (no 2020‐02593). Patients have received and signed written informed consent according to the instructions of the approval. All data were de‐identified following collection.

## 3 RESULTS

**Figure 1:**
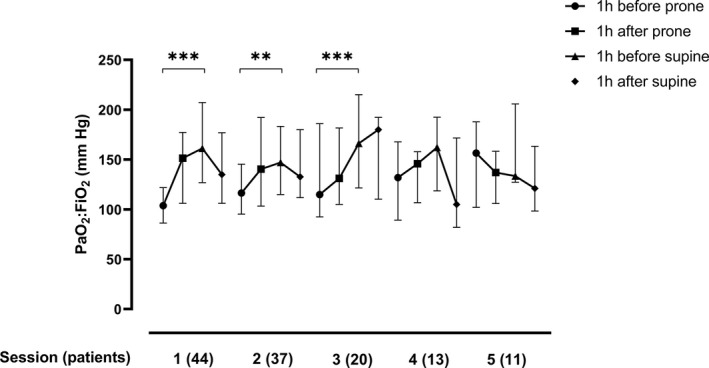
PaO_2_:FiO_2_ during five consecutive prone positioning sessions. Displayed as medians with IQR. ***P < .001, **P < .005

**Figure 2:**
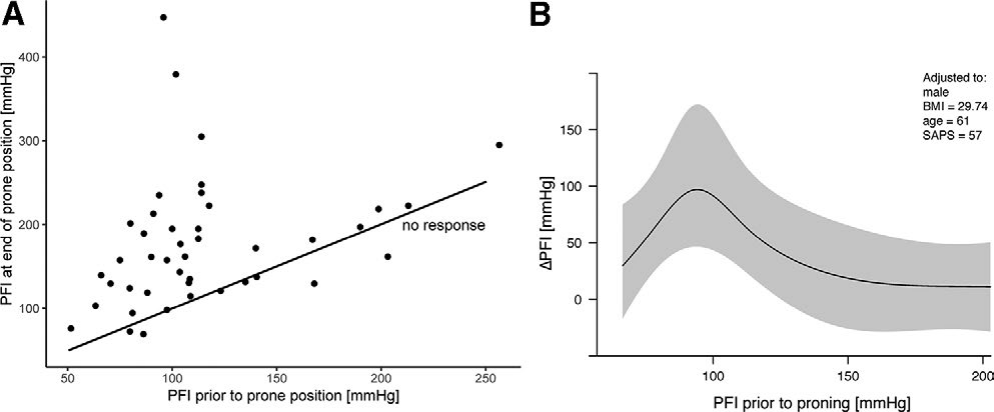
(a) PaO_2_:FiO_2_ (PFI) at the end of the first proning session as a function of the initial PaO_2_:FiO_2_. The line of no response is shown in black. (b) The predicted effect of initial PaO_2_:FiO_2_ (PFI) on the change in PaO_2_:FiO_2_ (ΔPFI) from the ordinal regression model, taken at the median values of the other covariables

In this cohort of mechanically ventilated patients in the ICU, we identified 44 patients who had been treated with prone positioning for a total of 138 sessions. The characteristics of the patients were (median (IQR)): age 62 (52‐69), SAPS 3, 58 (53‐61), days with symptoms before proning 13 (10‐24), and days in ventilator before proning 1 (1‐2). Seventy‐three percent were male. Most prevalent comorbidities were (N (%)): BMI > 30, 22 (50%), hypertension 21 (48%), diabetes mellitus 10 (23%), COPD/asthma 8 (18%), psychiatric disease 8 (18%), neurological disease 6 (14%), and heart disease 5 (11%). The number of consecutive proning was 1 to 8, with a median (IQR) of 2 (2‐4.25). The first five pronings for each patient were included, as the number of patients for proning 6‐8 was below 10. Parameters for the first session are displayed in Table 1. Parameters for sessions 2‐5 are provided in Supplemental Table 1. Median (IQR) time in prone positioning for the five sessions was 14 (12‐17) hours. In the first session, median (IQR) PaO_2_:FiO_2_ increased from 104 (86‐122) to 161 (127‐207) mm Hg (*P* < .001). A significant increase occurred in the first three sessions (Figure 1). Median (IQR) FiO_2_ decreased from 0.7 (0.6‐0.8) to 0.5 (0.35‐0.6) (<0.001). A significant decrease occurred in the first three sessions. PaO_2_ did not differ and PaCO_2_ decreased in the last session. Tidal volumes and PEEP did not differ. Mean arterial pressure (MAP) and norepinephrine infusion, pH, and base excess did not differ. Complications included 21 (48%) cases of facial edema, 18 (41%) cases of pressure sores, 11 (25%) cases of airway complication/ETT obstruction, and one (2%) case of nerve damage of the arm. At the 30‐day follow‐up, 12 (27.3%) patients were still admitted to the ICU, 20 (45.5%) patients were discharged from the ICU, and 12 (27.3%) patients had died. Improvement in PaO_2_:FiO_2_ was shown in 36 of 44 patients (82%). Responders to improved PaO_2_:FiO_2_ were patients with PaO_2_:FiO_2_ approximately < 120 mm Hg before the first proning session (Figure 2A). Age, sex, BMI, or SAPS 3 did not predict success in increasing PaO_2_:FiO_2_ (Figure 2B).

**Table 1:** Ventilatory, metabolic, and circulatory data from the first prone positioning session

Parameter. median (IQR)	1 h before prone	1 h after prone	1 h before supine	1 h after supine	*P*‐value
PaO_2_:FiO_2_ (mm Hg)	104 (86‐122)	151 (105‐178)	161 (127‐207)	135 (106‐177)	<.001
FiO_2_	0.7 (0.6‐0.8)	0.6 (0.5‐0.7)	0.5 (0.35‐0.6)	0.58 (0.45‐0.7)	<.001
Pao_2_ (mm Hg)	72 (65‐83)	78 (73‐95)	74 (68‐81)	71 (65‐77)	.49
Paco_2_ (mm Hg)	46 (41‐52)	48 (42‐54)	46 (41‐51)	46 (41‐54)	.50
Tidal volume (ml)	438 (376‐510)	445 (385‐513)	445 (400‐536)	450 (396‐490)	.46
Tidal volume (ml per kg of PBW)	6 (6‐8)	6 (6‐8)	6 (6‐9)	7 (6‐7)	.42
Respiratory frequency (breaths per min)	20 (17‐22)	20 (17‐25)	22 (17‐26)	22 (20‐25)	.07
PEEP (cm H2O)	11 (9‐12)	10 (10‐12)	10 (8‐11)	10 (9‐11)	.12
Plateau (cm H2O)	25 (21‐30)	25 (21‐28)	25 (21‐29)	24 (22‐29)	.75
Arterial pH	7.36 (7.33‐7.39)	7.35 (7.30‐7.38)	7.37 (7.33‐7.41)	7.37 (7.33‐7.40)	.12
Base Excess	1 (−2‐2.3)	0 (−2‐1.8)	1 (−1‐3)	1 (−1‐3)	.10
MAP (mm Hg)	75 (68‐82)	79 (70‐83)	77 (72‐82)	75 (70‐80)	.65
Norepinephrine (µg/kg/min)	0.06 (0.01‐0.1)	0.06 (0.02‐0.15)	0.05 (0.02‐0.15)	0.07 (0.03‐0.18)	.56
Time in prone position (h)			14.5 (13.0‐18.0)		

## 4 DISCUSSION

In this case series of mechanically ventilated COVID‐19 ARDS patients, we report that prone positioning had an 82% success rate of increased PaO_2_:FiO_2_, and that the effect was consistent after repeated prone positioning sessions. All COVID‐19 positive patients with severe ARDS, as defined according to the American‐European Consensus Conference criteria for severe ARDS (PaO_2_:FiO_2_ ratio of < 150 mm Hg, with a FiO_2_ of ≥ 0.6) ^10^ and who did not fulfil any exclusion criteria,^7^ were proned. Primarily, proning allowed for a decrease in FiO_2_, while PaO_2_ remained unchanged, which likely reflects the attending intensivist decreasing FiO_2_ in the ventilator as a response to improved PaO_2_. The improvement in oxygenation occurred independently of changes in ventilatory pressures and volumes, and proning was hemodynamically tolerable. Norepinephrine was the primary vasopressor to maintain adequate MAP and no patient required additional inotropic drugs.

All patients were proned within 1‐2 days after the initiation of mechanical ventilation. Guérin et al proned patients with ARDS who had been mechanically ventilated for less than 36 hours and concluded that early application of prolonged prone‐positioning sessions significantly decreased 28‐day and 90‐day mortality.^7^ The lung stiffness in ARDS lungs increases during mechanical ventilation,^11^ and it is possible that the time factor and ventilator days are of importance also in the proning of COVID‐19 patients. This is possibly the explanation to why the first three proning sessions were most successful in improving PaO_2_:FiO_2_, although the effect on mortality remains to be investigated.

Prone positioning may also cause potentially severe complications which should be weighed against the potential benefits of the procedure. The complications we report were higher in facial edemas and lower in airway obstruction compared to a previous study.^12^ Given the high complication rate of the procedure and no increase of PaO_2_:FiO_2_ after three sessions, it is possible that prone positioning after this time could do more harm than benefit.

The understanding of the heterogeneity of COVID‐19 ARDS (eg, pathophysiological features, clinical course, biomarkers, and phenotypes based on respiratory mechanics) is at an early stage. While the identification of phenotypes could ultimately help to guide the management of patients who are critically ill with COVID‐19, the emerging evidence of a similar pathophysiology of COVID‐19 ARDS suggest standard evidence‐based care. Patients with COVID‐19 ARDS present with a form of injury that, in many aspects, is similar to that of those with ARDS of other origins.^13^ In ARDS of other origins, and in the absence of contraindications, prone positioning should be considered in mechanically ventilated patients with PaO_2_:FiO_2_ < 150 mm Hg.^14^ This case series shows that prone positioning improved oxygenation in patients with severe COVID‐19 ARDS, and that responders to improved PaO_2_:FiO_2_ were patients with PaO_2_:FiO_2_ approximately < 120 mm Hg before the first proning session. Age, sex, BMI, or SAPS 3 did not predict success in increasing PaO_2_:FiO_2_. Thus, proning was effective in COVID‐19 ARDS, similarly to ARDS of other origins, and may be considered according to standard protocols for severe ARDS. In addition, the study serves as an indication of design of randomized trials to determine the effect on survival in patients with severe COVID‐19 ARDS. Limitations of the study include the small sample size, a short follow‐up time, and lack of a control group remaining in supine position.

## 5 CONCLUSION

Proning increased PaO_2_:FiO_2_, primarily in patients with PaO_2_:FiO_2_ approximately < 120 mm Hg, with a consistency over three sessions. No characteristic was associated with non‐responding, why proning may be considered in most patients. Further study is required to evaluate mortality.

## Supporting information

Table S1
