# Supplementary material for: Prone positioning in mechanically ventilated patients with severe acute respiratory distress syndrome and coronavirus disease 2019
Source: Acta Anaesthesiol Scand. 2020 Nov 22;65(3):360–3. doi: 10.1111/aas.13741 (PMC7894343; doi:10.1111/aas.13741)
Supplement: Supplementary file 1 — Table S1 [file AAS-65-360-s001.docx]

|  | **Session 2** |  |  |  |  | **Session 3** |  |  |  |  |
| --- | --- | --- | --- | --- | --- | --- | --- | --- | --- | --- |
| Parameter, median (IQR) | 1h before prone | 1h after prone | 1h before supine | 1h after supine | P-value | 1h before prone | 1h after prone | 1h before supine | 1h after supine | P-value |
| Pao2:Fio2 (mm Hg) | 117 (95-145) | 141 (103-192) | 147 (115-183) | 133 (112-180) | 0.0032 | 115 (93-186) | 131 (105-182) | 166 (122-215) | 180 (110-193) | <0.001 |
| Fio2 | 0.6 (0.53-0.7) | 0.55 (0.45-0.68) | 0.5 (0.43-0.6) | 0.55 (0.4-0.66) | 0.001 | 0.63 (0.43-0.7) | 0.6 (0.4-0.7) | 0.5 (0.38-0.58) | 0.55 (0.4-0.68) | 0.0045 |
| Pao2 (mm Hg) | 69 (65-80) | 76 (68-84) | 72 (69-80) | 72 (67-80) | 0.43 | 70 (59-80) | 74 (68-83) | 74 (69-84) | 76 (64-89) | 0.16 |
| Paco2 (mm Hg) | 47 (42-51) | 48 (41-55) | 45 (41-51) | 46 (41-50) | 0.69 | 47 (44-49) | 48 (42-51) | 48 (40-51) | 47 (44-54) | 0.07 |
| Tidal volume (ml) | 438 (409-480) | 442 (404-480) | 450 (405-480) | 450 (416-469) | 0.36 | 484 (427-524) | 470 (410-517) | 460 (420-500) | 433 (403-487) | 0.08 |
| Tidal volume (ml per kg of PBW) | 7 (6-7) | 6 (6-7) | 7 (6-7) | 7 (6-7) | 0.36 | 7 (6-8) | 7 (6-8) | 6 (6-8) | 6 (6-8) | 0.27 |
| Respiratory frequency (breaths per min) | 22 (20-25) | 22 (20-24) | 23 (20-26) | 22 (20-25) | 0.25 | 23 (15-26) | 20 (15-25) | 20 (14-25) | 23 (18-25) | 0.13 |
| PEEP (cm H2O) | 10 (9-12) | 10 (9-12) | 10 (9-12) | 10 (9-12) | 0.13 | 10 (9-12) | 10 (8-12) | 11 (10-14) | 11 (10-13) | 0.50 |
| Pplateau (cm H2O) | 25 (22-28) | 26 (21-29) | 26 (23-29) | 24 (21-27) | 0.85 | 25 (22-28) | 26 (23-29) | 27 (23-28) | 27 (24-29) | 0.42 |
| Arterial pH | 7.39 (7.35-7.42) | 7.37 (7.33-7.42) | 7.39 (7.36-7.42) | 7.39 (7.36-7.42) | 0.30 | 7.38 (7.34-7.42) | 7.38 (7.35-7.40) | 7.39 (7.33-7.43) | 7.38 (7.36-7.42) | 0.51 |
| Base Excess | 1.5 (0-5) | 2 (-1-4.5) | 3 (0-5.8) | 3 (0-5) | 0.08 | 2 (-1.8-5) | 2 (-1-5) | 2 (-0.3-5) | 3 (-1.5-5) | 0.36 |
| MAP (mm Hg) | 78 (73-83) | 83 (77-88) | 75 (70-81) | 77 (70-82) | 0.79 | 79 (73-82) | 80 (75-84) | 75 (70-81) | 77 (73-82) | 0.20 |
| Norepinephrine (µg/kg/min) | 0.07 (0.03-0.14) | 0.05 (0.03-0.13) | 0.05 (0.03-0.12) | 0.06 (0.04-0.13) | 0.31 | 0.06 (0.05-0.1) | 0.06 (0.02-0.1) | 0.03 (0.02-0.07) | 0.06 (0.02-0.12) | 0.10 |
| Time in prone position (h) |  |  | 15.0 (12.0-17.0) |  |  |  |  | 14.0 (12.0-18.5) |  |  |
|  |  |  |  |  |  |  |  |  |  |  |
|  | **Session 4** |  |  |  |  | **Session 5** |  |  |  |  |
| Parameter, median (IQR) | 1h before prone | 1h after prone | 1h before supine | 1h after supine | P-value | 1h before prone | 1h after prone | 1h before supine | 1h after supine | P-value |
| Pao2:Fio2 (mm Hg) | 132 (89-168) | 146 (107-158) | 162 (119-193) | 105 (82-172) | 0.25 | 157 (102-188) | 137 (106-159) | 133 (127-206) | 121 (98-163) | >0.99 |
| Fio2 | 0.58 (0.4-0.78) | 0.58 (0.45-0.73) | 0.5 (0.43-0.55) | 0.65 (0.45-0.83) | 0.13 | 0.53 (0.41-0.68) | 0.58 (0.48-0.64) | 0.5 (0.4-0.6) | 0.65 (0.45-0.75) | 0.88 |
| Pao2 (mm Hg) | 71 (64-76) | 74 (67-90) | 73 (66-83) | 69 (61-80) | 0.12 | 77 (68-82) | 74 (68-84) | 74 (71-95) | 74 (67-85) | >0.99 |
| Paco2 (mm Hg) | 44 (42-51) | 47 (44-52) | 47 (44-50) | 50 (45-54) | 0.65 | 50 (45-54) | 49 (46-55) | 45 (42-49) | 48 (43-58) | 0.03 |
| Tidal volume (ml) | 480 (426-510) | 484 (409-512) | 460 (412-525) | 415 (385-508) | 0.31 | 477 (396-489) | 444 (346-570) | 428 (289-501) | 420 (413-537) | 0.22 |
| Tidal volume (ml per kg of PBW) | 8 (6-9) | 8 (6-9) | 7 (6-8) | 7 (6-9) | 0.38 | 7 (6-9) | 7 (6-11) | 6 (6-8) | 7 (6-10) | 0.22 |
| Respiratory frequency (breaths per min) | 22 (15-25) | 20 (12-24) | 23 (19-24) | 21 (12-25) | 0.94 | 22 (20-27) | 23 (20-27) | 23 (17-24) | 21 (16-24) | 0.94 |
| PEEP (cm H2O) | 11 (10-11) | 10 (10-12) | 11 (10-12) | 10 (8-11) | >0.99 | 11 (9-12) | 10 (9-11) | 10 (10-12) | 10 (10-12) | >0.99 |
| Pplateau (cm H2O) | 25 (22-27) | 27 (24-28) | 25 (21-27) | 25 (21-29) | 0.52 | 24 (21-30) | 27 (21-30) | 26 (23-28) | 25 (24-29) | >0.99 |
| Arterial pH | 7.41 (7.35-7.43) | 7.38 (7.32-7.44) | 7.39 (7.35-7.42) | 7.38 (7.34-7.42) | 0.87 | 7.39 (7.37-7.42) | 7.39 (7.30-7.44) | 7.40 (7.39-7.44) | 7.37 (7.31-7.45) | 0.03 |
| Base Excess | 2.5 (-1.8-5.5) | 1 (-1.5-6.5) | 3.5 (0.3-7) | 2 (0-6.5) | 0.26 | 2 (0-7) | 2 (-2-7.5) | 6 (0.5-7.5) | 2 (-1-6.5) | 0.28 |
| MAP (mm Hg) | 77 (72-80) | 79 (74-84) | 78 (74-84) | 80 (75-83) | 0.45 | 77 (74-82) | 80 (74-84) | 81 (77-94) | 82 (75-83) | 0.23 |
| Norepinephrine (µg/kg/min) | 0.06 (0.04-0.09) | 0.04 (0.03-0.06) | 0.06 (0.01-0.1) | 0.04 (0.01-0.09) | 0.73 | 0.06 (0.02-0.08) | 0.06 (0.05-0.08) | 0.03 (0.02-0.08) | 0.05 (0.01-0.09) | 0.63 |
| Time in prone position (h) |  |  | 14.0 (10.5-14.5) |  |  |  |  | 14.0 (12.0-16.5) |  |  |
